# Experimental Analysis of Automatic Discrimination Performance Between Simulated Bruxism and Non‐Bruxism Under Conscious Conditions Using Electromyography and Machine Learning

**DOI:** 10.1155/ijod/7874254

**Published:** 2026-01-21

**Authors:** Hajime Minakuchi, Mitsuhiro Nagasaki, Lộc Hoàng Đình, Haruna Miki, Ko Omori, Tazuko Nishimura, Takuo Kuboki, Nobuaki Minematsu

**Affiliations:** ^1^ Department of Oral Rehabilitation and Regenerative Medicine, Okayama University Graduate School of Medicine, Dentistry and Pharmaceutical Sciences, Kita-ku, 700–8525, Okayama, Japan, okayama-u.ac.jp; ^2^ Department of Electrical Engineering and Information Systems, Graduate School of Engineering, The University of Tokyo, Tokyo, Japan, u-tokyo.ac.jp

**Keywords:** bruxism, dentistry, electromyography, EMG discrimination, machine learning

## Abstract

**Purpose:**

This study aimed to evaluate the potential use of machine learning to automatically classify electromyography (EMG) data into bruxism simulated movement with tooth contact (BMwTC), bruxism simulated movement without tooth contact (BMwoTC), and non‐bruxism movement (non‐BM).

**Methods:**

Twelve eligible healthy participants (female/male: 2/10, mean age: 35.3 ± 8.4 years) were asked to perform the simulated movements (all the tasks were performed five times for 5 s each with a 30‐s rest interval). The electrodes were placed on the masseter, infrahyoid, inframandibular, and chin muscles. A sound sensor was placed adjacent to the masseter. The EMG and sound data were sampled at 1 and 44.1 kHz, respectively. Single‐ and multi‐stream hidden Markov models (HMMs) were used to automatically discriminate the tested behavior from the others using a hamming window with 100 ms and shift length of 50 ms. The leave‐one‐out method was used for training and testing the model, with data from 11 participants used for training and one for testing. Each participant was evaluated, and the final performance was measured by averaging the results of 12 classification trials. The validity of the discrimination was assessed by calculating the harmony mean values using six EMG signals and the sound data.

**Results:**

The masseter EMG demonstrated significantly higher discrimination accuracy in the single‐stream model (*p*  < 0.05, One‐way ANOVA, Tukey HDS). The multi‐stream model also demonstrated higher accuracy; however, no significant difference was observed. Notably, the accuracy of BMwoTC was less than 0.5.

**Conclusion:**

The machine‐learning‐based discriminative system accurately discriminates BMwTC from non‐BM using masseter EMG.

## 1. Introduction

According to “International consensus on the assessment of bruxism (2018)”, the definition of sleep bruxism (SB) is masticatory muscle activity during sleep that is characterized as rhythmic (phasic) or non‐rhythmic (tonic) and is not a movement disorder or a sleep disorder in otherwise healthy individuals. Furthermore, awake bruxism is defined as masticatory muscle activity during wakefulness characterized by repetitive or sustained tooth contact and/or bracing or thrusting of the mandible, and it is not a movement disorder in otherwise healthy individuals [1]. They also concluded that bruxism should not be considered a disorder but rather a behavior that can be a risk (and/or protective) factor for certain clinical consequences in otherwise healthy individuals.

On the other hand, the case of SB represents in excessive occlusal forces greater than the maximum clenching force [2]; therefore, specific SB would be considered an aggravating factor for tooth, periodontal tissue, masticatory muscle, and temporomandibular joint disorder [3]. Ramfjord was the first to objectively describe the SB behavior of participants with psychiatric diseases in detail using electromyography (EMG) [4]; polysomnography (PSG) was subsequently employed to measure other physiological factors concerning SB [5]. Since these studies demonstrated the hyperactivity of the masticatory muscle to be a coexisting symptom of bruxism, EMG hyperactivity has become the standard parameter for detecting the presence of bruxism. However, several limitations of EMG‐based SB assessments have recently been reported. Specifically, head/body movements, swallowing, and other nonspecific movements can be erroneously detected as SB episodes, especially in single‐channel EMG assessments [6, 7]. Single‐channel EMG is delivered as a small ambulatory all‐in‐one device (EMG logger; GC, JAPAN) with low physical or mental burden [8]. However, the validity of the current assessment system, which only counts the number of EMG episodes that exceed a certain threshold and does not examine the details of the EMG data, is questionable [9, 10].

Recently, bracing, defined as the forceful maintenance of a specific mandibular position, has been introduced as a subcategory of bruxism [1, 11]; no tooth contact occurs during bracing despite the hyperactivity of the jaw opening and closing muscles. Bracing does not have a detrimental effect on the teeth, but rather on the temporomandibular joint and masticatory muscles [1, 12]. In contrast, clenching affects the occlusal hyper‐mechanical stress on the teeth. Therefore, it is important to assess the mechanical stress on the teeth to differentiate between bracing and clenching. However, distinguishing between bracing and clenching using EMG‐based assessment may be challenging, as both events exhibit hyperactivity of the jaw‐closing muscles [13]. Moreover, assessing the presence of tooth contact by extraoral observation is also complicated. Therefore, a novel detection method differentiating between clenching and bracing is necessary.

Machine learning is expected to offer solutions to this problem. Automatic speech recognition (ASR) exemplifies a machine learning technique used to extract linguistic information from speech waveforms [14]. For instance, identifying phonemes and words visually, by human examiners, is extremely challenging, but ASR can easily identify these components within speech waveforms. ASR extracts useful features from speech signals to determine linguistic content. This approach can also be applied to EMG waveform data, as it contains valuable features. Therefore, this machine learning‐based automatic detection system might be capable of distinguishing EMG data associated with various types of bruxism. However, the validity of this system still needs to be verified. Consequently, this study aims to test whether machine learning methods can help identify EMG data indicating bruxism‐like and non‐bruxism‐like behaviors.

## 2. Materials and Methods

### 2.1. Participants

Participants were recruited from the Okayama University Graduate School of Medicine, Dentistry, and Pharmaceutical Sciences faculty members and clinical staff at Okayama University Hospital. Participants were excluded if they (1) were receiving orthodontic treatment; (2) took psychotropic medication, hypnotic medication, muscle relaxants, or antidepressants; or had any prescription of medicine or drugs maintaining possible sleep effect or possibly altering the sleep behavior within the past 6 months; (3) had been diagnosed with a cutaneous problem (e.g., atopic dermatitis, allergy to electrode gel); (4) were pregnant; (5) abused alcohol or consumed more than three cups of coffee per day; (6) had more than two missing posterior teeth, not including the third molars; or (7) wore a removable prosthesis (partial or complete dentures). Twelve eligible participants signed a consent form after eligibility screening.

Conversely, participant data were only accepted if the EMG channel signals, audio, or video recordings were obtained entirely during scoring. None of the participants withdrew from the study or hesitated to sleep in the sleep laboratory. All the included participants had average weight or were mildly overweight, and were generally healthy adults with no diagnosed oral para‐functional habits, systemic neuromuscular disorders, or temporomandibular disorders. The study protocol was approved by the Ethics Committee of the Okayama University Graduate School of Medicine, Dentistry and Pharmaceutical Science (RIN #2008‐003).

### 2.2. EMG Data and Skin Surface Sound Collection

Each participant underwent EMG assessment in an audio‐visually monitored, dark, partially soundproof, temperature‐controlled recording room. EMG recordings were performed at an accredited sleep laboratory (Okayama University Hospital, Okayama, Japan), and multiple EMG recordings were obtained using the PSG1100 (Nihon Kohden, Japan) system. The electrode used in this study was a disk monopolar electrode set (H503A, NE‐113A, Nihon Kohden, Japan) with an impedance of 100 MΩ. The following observations were made: (1) bilateral masseter, (2) bilateral infrahyoid muscle, (3) chin, and (4) right suprahyoid muscle EMG, and (5) cutaneous transmittance sound on the right cheek recorded using a small microphone‐inserted stethoscope. The sampling frequency of the EMG was set to 1000 Hz and that of the sound data to 16 kHz, which was translated to 44.1 kHz. The high/low‐pass filter was set at 10 Hz/500 Hz, respectively, for the EMG recording.

### 2.3. Experimental Task

**Figure 1:**
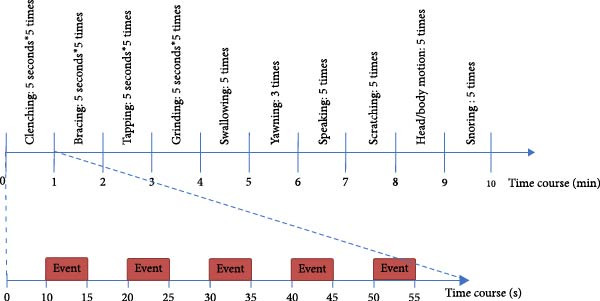
Experimental tasks and procedures.

Before attaching the electrodes, oil from the participant’s face was removed using a skin preparation gel. After setting up the electrodes, the participants were placed in a dorsal position to replicate the sleep condition on the bed in the sleep laboratory and were asked to perform the experimental tasks. Each task consisted of five series of movements with a 5s interval between two consecutive series, and the rest time between the tasks was set to 30 s. The same researcher instructed the participants on how to perform these tasks using the established protocol. The experimental tasks included 100% maximal voluntary contraction (MVC) clenching, bracing (100% MVC clenching without tooth contact), tapping, grinding, swallowing, yawning, speaking, facial scratching, head/body motion, and snoring. The participants were instructed to perform a bracing behavior involving sustained contraction of both the jaw opening and closing muscles without tooth contact or mandibular movement. The recording procedure is illustrated in Figure 1 and Table 1. Clenching, grinding, and tapping were categorized as simulated bruxism movements with tooth contact (BMwTC); swallowing, yawning, speaking, scratching the face, head/body motion, and snoring were categorized as simulated non‐bruxism movements (non‐BMs), and bracing was categorized as simulated bruxism movements without tooth contact (BMwoTC).

**Table 1:** Details of experimental tasks.

Experimental task	Category	Descriptions
Clenching	BMwTC	Biting or holding the tooth contacting hardly for a while without lower jaw movement
Bracing	BMwoTC	Bracing the jaw hardly without jaw movement and tooth contacting
Tapping	BMwTC	Biting the upper and lower teeth several times but not for functional
Grinding	BMwTC	The lower jaw tooth is tightened, rubbed against the upper jaw tooth side to side, creating a typical grinding sound
Swallowing	non‐BM	Usual act of swallowing the saliva without food
Yawning	non‐BM	An opening of mouth wide to take a deep breath
Speaking	non‐BM	Talking during the experiment
Scratching	non‐BM	Scraping or touch the skin of the face or head
Head/body motion	non‐BM	Body movement during experiment
Snoring	non‐BM	Make the snoring

Abbreviations: BMwoTC, bruxism movement without tooth contact; BMwTC, bruxism movement with tooth contact; non‐BM, non‐bruxism movement.

### 2.4. Machine Learning

Currently, several deep learning models are widely applied in the diagnostic sciences field [15]. A hidden markov model (HMM) is applied in this study to model temporal sequences of feature vectors for a specific class [16, 17]. An HMM explicitly models “time‐sequential state transitions” by using transition probabilities between states at each point in time and the next state, making the temporal dependency very clear. In contrast, convolutional neural networks (CNN) excel at extracting image and spatial features but cannot directly express temporal dependencies. For this reason, an HMM was used in this study. After training the HMMs for the individual classes, they can be used to classify any input vector(s) into one of the classes. Spectrum‐based features, such as the Mel frequency cepstrum coefficient (MFCC), are commonly used in ASR to effectively characterize different phonemes. Therefore, this study also applied MFCCs as features. The MFCCs were calculated from electromyographic signals of conscious bruxism/non‐bruxism data using a Hamming window of 100 ms length and a 50 ms shift. The hum noise caused by the power supply was eliminated using a hum filter. Thirteen dimensions of the MFCC, including the 0th order, were computed using ∆ features which represent the temporal dynamics or velocity components of the MFCC [18, 19].

A three‐state single‐stream HMM with an output probability Gaussian distribution of 10 mixtures was used in this study using MATLAB (R2023a, MathWorks Inc., Natick, MA, USA) and the Statistics and Machine Learning Toolbox. Specifically, three classes—BMwTC, non‐BM, and BMwoTC—were modeled separately as three different HMMs using the MFCC features. In addition to the previous single‐stream HMM, a multi‐stream HMM, which could simultaneously process multiple features, such as masseter EMG, infrahyoid muscle EMG, and cutaneous transmittance sounds using MFCC features, was applied in this study using MATLAB (R2023a, MathWorks Inc., Natick, MA, USA) and the Statistics and Machine Learning Toolbox [20]. Thus, the validity of this multi‐stream HMM was evaluated by separating the three classes: BMwTC, non‐BM, and BMwoTC. The biological sources of the multi‐stream analysis model were: (1) EMG of the bilateral masseter, bilateral infrahyoid muscle, chin and right suprahyoid muscles (MS‐1), (2) EMG of the bilateral masseter muscles (MS‐2), (3) EMG of the bilateral masseter muscles and cutaneous transmittance sound (MS‐3), (4) EMG of the bilateral masseter and bilateral infrahyoid muscles (MS‐4), and (5) bilateral masseter and bilateral infrahyoid muscles and cutaneous transmittance sound (MS‐5).

### 2.5. Data Analysis and Statistical Analysis

**Figure 2:**
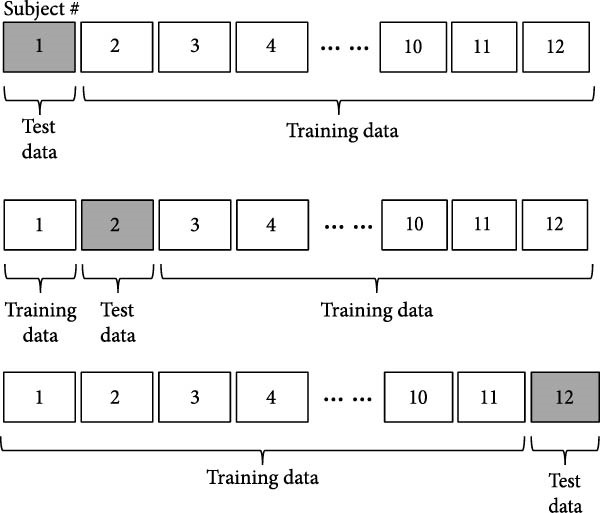
Leave one out methods.

The leave‐one‐out method, where data from one participant are used to test the HMM, was trained with data from the other 11 participants. By using each of the 12 participants as test subjects and averaging the identification performance across the 12 experiments, we obtained the overall performance of our HMM (Figure 2). The overall performance of the models was calculated based on the features derived from each class of the training data, utilizing the following metrics: sensitivity, specificity, and positive and negative predictive values. Additionally, the harmonic mean value (*F* value = [2 × positive predictive value × sensitivity]/[positive predictive value + sensitivity]) was calculated for each model, and the overall accuracy was assessed. Statistical analysis was conducted to compare the mean *F*‐value between the single‐stream model and the multi‐stream model using One‐way factorial ANOVA. Furthermore, the highest parameter was compared using a post hoc test (Tukey HDS). A significance level of *α* = 0.05 was established.

## 3. Results

### 3.1. Single‐Stream HMM

A total of 12 participants (female/male: 2/10; mean age = 35.3 ± 8.4 years) underwent EMG examination. The mean sensitivity, specificity, and positive and negative predictive values for detecting non‐BM and BMwoTC among six EMG signals and one sound dataset are listed in Tables 2–4. Among these, the bilateral masseter demonstrated the highest accuracy in detecting both BMwTC and non‐BM; the corresponding *F*‐values were 0.77 ± 0.08 and 0.77 ± 0.13 for BMwTC, and 0.91 ± 0.04 and 0.87 ± 0.05 for non‐BM, respectively. Conversely, the EMG of the suprahyoid muscles displayed the highest sensitivity, specificity, and positive and negative predictive values in detecting BMwoTC among the seven parameters; however, the mean *F*‐value was 0.35 ± 0.23. Additionally, the specificity and negative predictive value for BMwoTC detection were above 0.8 for all seven parameters. The mean *F*‐values exhibited a statistically significant difference among these seven parameters regarding BMwTC and non‐BM (Table 5). Notably, the results of the post hoc test indicated that the masseter EMG and sound data were significantly higher than all other parameters concerning BMwTC (*p*  < 0.05, one‐way factorial ANOVA, Tukey HSD (Tables 6 and 7).

**Table 2:** Validity of the BMwTC detection in single‐stream HMM.

	Sensitivity	Specificity	Positive predictive value	Negative predictive value
Right masseter	0.81 ± 0.14	0.95 ± 0.03	0.76 ± 0.12	0.96 ± 0.03
Left masseter	0.78 ± 0.22	0.95 ± 0.03	0.77 ± 0.11	0.96 ± 0.04
Right infrahyoid muscles	0.62 ± 0.31	0.42± 0.18	0.15 ± 0.06	0.88 ± 0.08
Left infrahyoid muscles	0.59 ± 0.35	0.46 ± 0.19	0.16 ± 0.07	0.88 ± 0.08
Chin	0.50 ± 0.24	0.83 ± 0.10	0.38 ± 0.15	0.90 ± 0.04
Suprahyoid muscles	0.64 ± 0.22	0.64 ± 0.13	0.25 ± 0.07	0.91 ± 0.04
Sound data	0.66 ± 0.26	0.91 ± 0.06	0.61 ± 0.15	0.94 ± 0.04

*Note:* mean ± SD.

**Table 3:** Validity of the BMwoTC detection in single‐stream HMM.

	Sensitivity	Specificity	Positive predictive value	Negative predictive value
Right masseter	0.47 ± 0.39	0.90 ± 0.05	0.18 ± 0.15	0.97 ± 0.02
Left masseter	0.45 ± 0.41	0.86 ± 0.08	0.11 ± 0.09	0.97 ± 0.02
Right infrahyoid muscles	0.67 ± 0.37	0.85 ± 0.09	0.17 ± 0.11	0.98 ± 0.02
Left infrahyoid muscles	0.60 ± 0.43	0.82 ± 0.15	0.15 ± 0.13	0.98 ± 0.02
Chin	0.25 ± 0.26	0.87 ± 0.12	0.10 ± 0.13	0.96 ± 0.02
Suprahyoid muscles	0.60 ± 0.40	0.93 ± 0.04	0.27 ± 0.20	0.98 ± 0.02
Sound data	0.45 ± 0.34	0.92 ± 0.05	0.19 ± 0.13	0.97 ± 0.02

*Note:* mean ± SD.

**Table 4:** Validity of the non‐BM detection in single‐stream HMM.

	Sensitivity	Specificity	Positive predictive value	Negative predictive value
Right masseter	0.86 ± 0.07	0.88 ± 0.14	0.97 ± 0.04	0.63 ± 0.11
Left masseter	0.81 ± 0.11	0.85 ± 0.21	0.96 ± 0.05	0.58 ± 0.16
Right infrahyoid muscles	0.25 ± 0.22	0.81 ± 0.30	0.91± 0.10	0.20 ± 0.07
Left infrahyoid muscles	0.27 ± 0.16	0.80 ± 0.19	0.88 ± 0.08	0.22 ± 0.04
Chin	0.72 ± 0.19	0.70 ± 0.24	0.91 ± 0.06	0.43 ± 0.13
Suprahyoid muscles	0.58 ± 0.15	0.80 ± 0.20	0.94 ± 0.05	0.33 ± 0.06
Sound data	0.86 ± 0.10	0.82 ± 0.19	0.95 ± 0.05	0.63 ± 0.14

*Note:* mean ± SD.

**Table 5:** Mean *F*‐value of BMwTC, BMwoTC, and non‐BM detection in single‐stream HMM.

	BMwTC	BMwoTC	Non‐BM
Right masseter	0.77 ± 0.08	0.23 ± 0.18	0.91 ± 0.04
Left masseter	0.77 ± 0.13	0.17 ± 0.15	0.87 ± 0.05
Right infrahyoid muscles	0.24 ± 0.10	0.27 ± 0.17	0.34 ± 0.22
Left infrahyoid muscles	0.24 ± 0.10	0.23 ± 0.19	0.39 ± 0.19
Chin	0.40 ± 0.13	0.14 ± 0.14	0.79 ± 0.14
Suprahyoid muscles	0.36 ± 0.09	0.35 ± 0.23	0.70 ± 0.10
Sound data	0.60 ± 0.16	0.25 ± 0.18	0.90 ± 0.05

*p*‐value	<0.01	0.10	0.00

*Note:* One‐way factorial ANOVA (mean ± SD).

**Table 6:** Results of post hoc test among the mean *F*‐value of BMwTC.

	Right masseter	Left masseter	Right infrahyoid muscles	Left infrahyoid muscles	Chin	Suprahyoid muscles	Sound data
Right masseter	—	1.00	<0.01	<0.01	<0.01	<0.01	<0.01
Left masseter		—	<0.01	<0.01	<0.01	<0.01	0.03
Right infrahyoid muscles			—	1.00	0.03	0.23	<0.01
Left infrahyoid muscles				—	0.03	0.20	<0.01
Chin					—	0.98	<0.01
Suprahyoid muscles						—	<0.01
Sound data							—

*Note: p*‐value, Tukey HSD.

**Table 7:** Results of post hoc test among the mean *F*‐value of non‐BM.

	Right masseter	Left masseter	Right infrahyoid muscles	Left infrahyoid muscles	Chin	Suprahyoid muscles	Sound data
Right masseter	—	0.99	<0.01	<0.01	0.27	0.01	1.00
Left masseter		—	<0.01	<0.01	0.67	0.04	1.00
Right infrahyoid muscles			—	0.97	<0.01	<0.01	<0.01
Left infrahyoid muscles				—	<0.01	<0.01	<0.01
Chin					—	0.74	0.35
Suprahyoid muscles						—	0.01
Sound data							—

*Note: p*‐value, Tukey HSD.

### 3.2. Multi‐Stream HMM

The accuracies of BMwTC and non‐BM in the multi‐stream HMM model tended to be higher than those in the single‐stream HMM model. Among the five types of MS models, these *F*‐values showed no significance even in the BMwTC, BMwoTC, and non‐BM groups, respectively (*p* = 0.31, 0.17, 0.10). Of the five models, MS‐5 demonstrated the highest sensitivity, specificity, and positive and negative predictive values in both BMwTC and non‐BM; the corresponding *F*‐values were 0.83 ± 0.09 in BMwTC and 0.94 ± 0.03 in non‐BM (Tables 8–11). Conversely, BMwoTC showed the lowest mean *F*‐value compared to BMwTC and non‐BM, with MS‐5 at 0.43 ± 0.25, and the others less than 0.5.

**Table 8:** Validity of the BMwTC detection in multi‐stream HMM.

	Sensitivity	Specificity	Positive predictive value	Negative predictive value
MS‐1	0.85 ± 0.16	0.96 ± 0.03	0.81 ± 0.11	0.97 ± 0.03
MS‐2	0.82 ± 0.16	0.96 ± 0.03	0.80 ± 0.11	0.97 ± 0.03
MS‐3	0.81 ± 0.16	0.96 ± 0.02	0.79 ± 0.09	0.97 ± 0.03
MS‐4	0.83 ± 0.22	0.96 ± 0.03	0.83 ± 0.09	0.97 ± 0.04
MS‐5	0.86 ± 0.16	0.96 ± 0.03	0.83 ± 0.10	0.97 ± 0.03

*Note:* mean ± SD. MS‐1: EMG of bilateral masseter muscle, bilateral infrahyoid muscle, chin and right suprahyoid muscle. MS‐2: EMG of bilateral masseter muscle. MS‐3: EMG of bilateral masseter muscle and cutaneous transmittance sound. MS‐4: EMG of bilateral masseter muscle and bilateral infrahyoid muscle. MS‐5: EMG of bilateral masseter muscle, bilateral infrahyoid muscle and cutaneous transmittance sound.

**Table 9:** Validity of the BMwoTC detection in multi‐stream HMM.

	Sensitivity	Specificity	Positive predictive value	Negative predictive value
MS‐1	0.58 ± 0.41	0.93 ± 0.04	0.23 ± 0.15	0.98 ± 0.02
MS‐2	0.45 ± 0.40	0.90 ± 0.06	0.17 ± 0.15	0.97 ± 0.02
MS‐3	0.52 ± 0.40	0.91 ± 0.05	0.18 ± 0.14	0.98 ± 0.02
MS‐4	0.66 ± 0.39	0.90 ± 0.06	0.23 ± 0.15	0.98 ± 0.02
MS‐5	0.58 ± 0.36	0.96 ± 0.03	0.37 ± 0.22	0.98 ± 0.02

*Note:* mean ± SD. MS‐1: EMG of bilateral masseter muscle, bilateral infrahyoid muscle, chin and right suprahyoid muscle. MS‐2: EMG of bilateral masseter muscle. MS‐3: EMG of bilateral masseter muscle and cutaneous transmittance sound. MS‐4: EMG of bilateral masseter muscle and the bilateral infrahyoid muscle. MS‐5: EMG of bilateral masseter muscle, bilateral infrahyoid muscle and cutaneous transmittance sound.

**Table 10:** Validity of the non‐BM detection in multi‐stream HMM.

	Sensitivity	Specificity	Positive predictive value	Negative predictive value
MS‐1	0.88 ± 0.06	0.85 ± 0.17	0.96 ± 0.04	0.67 ± 0.10
MS‐2	0.87 ± 0.07	0.87 ± 0.16	0.96 ± 0.04	0.65 ± 0.13
MS‐3	0.87 ± 0.07	0.87 ± 0.16	0.96 ± 0.04	0.66 ± 0.13
MS‐4	0.85 ± 0.07	0.86 ± 0.17	0.96 ± 0.04	0.62 ± 0.11
MS‐5	0.92 ± 0.06	0.84 ± 0.17	0.96 ± 0.04	0.76 ± 0.12

*Note:* mean ± SD. MS‐1: EMG of bilateral masseter muscle, bilateral infrahyoid muscle, chin and right suprahyoid muscle. MS‐2: EMG of bilateral masseter muscle. MS‐3: EMG of bilateral masseter muscle and cutaneous transmittance sound. MS‐4: EMG of bilateral masseter muscle and the bilateral infrahyoid muscle. MS‐5: EMG of bilateral masseter muscle, bilateral infrahyoid muscle and cutaneous transmittance sound.

**Table 11:** *F*‐value of BMwTC, BMwoTC, and non‐BM detection in multi‐stream HMM.

	BMwTC	BMwoTC	Non‐BM
MS‐1	0.82 ± 0.09	0.32 ± 0.21	0.92 ± 0.03
MS‐2	0.79 ± 0.09	0.22 ± 0.18	0.91 ± 0.03
MS‐3	0.79 ± 0.07	0.26 ± 0.21	0.92 ± 0.03
MS‐4	0.80 ± 0.15	0.33 ± 0.20	0.90 ± 0.04
MS‐5	0.83 ± 0.09	0.43 ± 0.25	0.94 ± 0.03

*p*‐value	0.31	0.17	0.10

*Note:* One‐way factorial ANOVA (mean ± SD). MS‐1: EMG of bilateral masseter muscle, bilateral infrahyoid muscle, chin and right suprahyoid muscle. MS‐2: EMG of bilateral masseter muscle. MS‐3: EMG of bilateral masseter muscle and cutaneous transmittance sound. MS‐4: EMG of bilateral masseter muscle and bilateral infrahyoid muscle. MS‐5: EMG of bilateral masseter muscle, bilateral infrahyoid muscle and cutaneous transmittance sound.

## 4. Discussion

This is the first study to evaluate the validity of an EMG‐based machine learning approach for simulated bruxism movements with tooth contact, non‐BMs, and bracing behavior. In addition, the novelty of this study lies in the comparison of different machine learning assessment models, such as single‐ and multi‐stream models. The single‐stream model indicated that the masseter EMG had the highest accuracy. The multi‐stream model suggested that the combination of the infrahyoid muscle EMG and cutaneous transmittance sound data would be helpful in increasing the detection accuracy compared to the single‐stream model.

Currently, the criteria for an SB event are assessed based on the amplitude of an EMG burst or an episode. Specifically, the American Academy of Sleep Medicine criteria and several recent studies have applied the “twice the baseline amplitude” criteria to the threshold of waveform amplitude of SB episodes, which have also been applied to the detection of SB episodes [9, 10, 21]. Regarding the number of SB bursts, a recent study demonstrated that the number of bursts assessed by single‐channel EMG with wave form thresholds of relatively low amplitude (e.g., twice the baseline or 5% MVC) had a better correlation with the number of SB episodes assessed by PSG plus audio and visual recording [10]. The SB detection methods used in this study were completely different from those used in previous amplitude‐based assessments, which focused on the features of SB EMG.

The HMM was applied to analyze the EMG waveform in this study. An HMM is composed of a left‐to‐right sequence of states, each of which is allowed to transition to the next state or its state, that is, a loop transition. By applying the HMM of each class to the MFCC feature vectors of the input EMG waveform, the HMM calculates the likelihood score of the feature vectors generated from each class. These scores were compared and used to determine the class of input EMG waveforms. The Viterbi score was used to identify BMwTC, non‐BM, and BMwoTC. This HMM probabilistically analyzes the characteristics of the EMG waveforms to detect using compatibility with the teacher data. Thus, the recognition performance may improve with a large amount of training data.

Single‐channel, amplitude‐based EMG systems have two major limitations. One is the lack of discrimination between awake and asleep conditions; thus, differentiating between hyperactivity of the muscles during sleep and awake conditions is challenging [9]. A recent study showed that the application of a modified cutoff value was helpful in diagnosing SB and compensated for this limitation, even when using single‐channel EMG [21]. However, if the sleep duration itself is questionable, the number of SB bursts and/or episodes per hour of sleep may also be unreliable. Owing to the inability to discriminate between SB events during sleep and nonspecific movements before sleep onset, the current system records the onset and end of sleep. Meanwhile, machine learning systems may be able to differentiate between the characteristics of sleep or wakefulness of the SB event itself; therefore, this limitation can be resolved. Thus, the validity of the number of sleep SBs might be higher than in conventional assessments.

Another limitation is the inability to discriminate between true and nonspecific SB movements. In the sleep data, the EMG bursts comprised both SB events and nonspecific movements, such as head movements, scratching, swallowing, snoring, and yawing. Differentiating between true SB and oromotor activities using amplitude‐and/or wave‐type‐based scoring systems would be challenging. Nevertheless, this study suggests that a machine‐learning system may be able to discriminate between true SB and oromotor activities with high accuracy. Thus, a machine learning system may overcome the limitations of the current SB assessment system.

On the other hand, this study had several limitations. First, this study obtained conscious simulated bruxism and non‐BMs as teacher data and evaluated the ability to discriminate between “simulated BM” and “simulated non‐BM.” Thus, we did not evaluate whether these simulated bruxism/non‐BMs would provide adequate teacher data for actual sleep behaviors. Since there may be a difference between the EMG of simulated unconscious bruxism movements and actual bruxism movements during sleep. Therefore, this point should be addressed in future studies aimed at evaluating whether intentionally conscious SB data are adequate as teacher data to differentiate between SB and non‐SB EMG. The validation of simulated BMwTC/BMwoTC/non‐BM will also be conducted in the future study using the real sleep EMG/PSG data. However, treating sleep data as teacher data is not feasible; thus, sleep data should be modified through an analytical process. Additionally, this study indicated a slightly higher *F*‐value in the bilateral masseter EMG model compared to the unilateral one. Therefore, the bilateral EMG model would be adequate; however, the feasibility of bilateral measurement may be questionable. Thus, this point should also be discussed in future studies. Second, the characteristics of the study participants were similar and gender‐imbalance. The characteristics of the EMG waveforms may vary according to generation, dentition, and sex; therefore, further analysis using different generations and dentition participant groups is necessary to improve the accuracy and feasibility before practical application. Third, this study only conducted a comprehensive evaluation of discrimination accuracy between “simulated bruxism movements” and “simulated non‐BMs.” Moreover, the accuracy of machine learning systems for each bruxism movement has not yet been clarified. This exploratory study aimed to determine whether machine learning could discriminate between SB movement group and non‐SB movement group in EMG wave data. Therefore, further studies are warranted to evaluate the discrimination ability of each subtype of SB and non‐SB movement in addition to apply the other advanced classifiers such as decision trees, support vector machines, CNN, or recurrent neural networks. Furthermore, electromyograms during bruxism were sampled in this study using simulated movements under conscious control. However, it is essential to note that the characteristics of simulated bruxism data during consciousness and bruxism data during actual sleep may not necessarily be the same. Future research should examine these differences, establish analytical methods using PSG‐based real sleep data, and clarify discrimination abilities. Fourth, as there was a silent period before the occurrence of the simulated SB/non‐SB events, the onset time of each event was reliably determined in this experimental study; however, in real sleep, multiple movements usually occur sequentially, which is a critical point. Future studies should focus on determining the reliability and accurate detection of the onset point for each movement. Fifth, the sample size of this study might not suffice to achieve the appropriate statistical power. Typically, sample size estimation should be performed before starting the study to determine the number of subjects based on previous study results. Nevertheless, this study is the first to apply machine learning analysis to differentiate between masticatory muscle activities. Therefore, no prior data was obtained, making sample size estimation impossible. Thus, it is unclear whether the sample size of this study is adequate or not, and consequently, the validity of this study’s results might also be uncertain. Furthermore, to elevate generalizability, the properties of subjects (e.g., age, gender, dental health condition) should be synchronized with those of the general population.

This study compared the discrimination accuracy of seven biological information items (six EMG signals and skin transmission sound). Among them, the masseter EMG showed the highest sensitivity, specificity, and positive and negative predictive values in single‐stream HMM. Furthermore, combining masseter muscle EMG with skin transmittance sound data increased detection accuracy in multi‐stream HMM; however, this was only observed for BMwTC and non‐BM. BMwoTC still showed low discrimination accuracy even in the MS model. The specific finding regarding discrimination ability in BMwoTC is that it has low sensitivity and a low positive predictive value. In BMwoTC, simulated as bracing, muscle activity increases without tooth grinding or tapping sound being generated, as occurs during clenching. This may make it difficult to distinguish between BMwoTC and clenching movements. In the future, it will be necessary to consider new methods for differentiating between bracing and clenching behavior.

Obstructive sleep apnea syndrome (OSAS) and gastroesophageal reflux disease (GERD) are reportedly associated with the occurrence of SB [22–25]; therefore, we included the EMG data of the swallowing and respiratory muscles. In particular, the suprahyoid muscle EMG showed moderate accuracy in detecting non‐BM in single‐stream HMM. Generally, the suprahyoid and infrahyoid muscles contribute to jaw movement during chewing, bolus formation, swallowing, and neck movements; thus, these EMG data would be helpful in increasing the accuracy of detecting the jaw‐opening related movements [26–28]. In fact, in the multi‐stream model, the model that included EMG from the suprahyoid and infrahyoid muscles demonstrated higher detection accuracy than the single‐stream model of the masseter muscle. However, the suprahyoid muscles are easily affected by head motion artifacts, and maintaining electrode stability is challenging. Therefore, obtaining reliable results in the supine position may be demanding with low feasibility.

However, tooth contact sound recorded by a stethoscope microphone is a clear indication of tooth contact [29]. Therefore, we included cutaneous transmission sounds as biological sources, and evaluated their usefulness. This study demonstrated that the cutaneous transmittance sound data had high specificity and negative predictive value, and the multi‐stream model including sound data had the higher detection accuracy compared to the single‐stream model. Thus, if the sound data indicate “positive” (presence of tooth sound), these participants would most likely have positive (tooth contacting) readings. This may be because tooth contact sounds, particularly tooth tapping and grinding sounds, which have specific waveforms, can provide reliable evidence of tooth contact. Unfortunately, a detailed analysis of the tooth‐contacting sound was not performed in this study; therefore, the validation remains unclear. Future research would need to analyze the details of the properties of tooth contacting sounds regarding the interval, maximum value, and properties of frequency.

However, it is still unclear whether tooth contact sounds can be detected during clenching behavior, and further research is warranted to determine methods for detecting clenching behavior. However, sound data may be useful complementary biological information to determine the presence of tooth contact that is not clearly detected by EMG, at least by tapping and grinding, which could not be detected by extraoral observation. Additionally, it would be interesting to analyze the performance of automatic discrimination with recently introduced features, such as smartphone applications and artificial intelligence, to understand its potential role in daily clinical practice [30, 31].

In prosthodontics, excessive mechanical stress worsens the prognosis of both teeth and prostheses. Therefore, detecting bruxism involving tooth contact is crucial for prognostic assessment. This study demonstrated low accuracy in detecting muscle hyperactivity without tooth contact sounds, suggesting that identifying bracing is challenging. Detecting tooth contact, in addition to muscle activity, is thus essential for evaluating mechanical stress. The multi‐stream model in this study also showed low accuracy in detecting bracing, highlighting the need to investigate the causes of this limitation. Because a single EMG assessment is insufficient for tooth contact detection, developing a novel, highly accurate assessment system is warranted. A machine learning‐based approach using EMG and additional biological signals, such as tooth contact sounds, may improve accuracy. However, further research is needed to clarify the discriminative ability among bruxism subtypes and non‐bruxism behaviors, and to validate the feasibility of data collection during sleep, applicability to different dentitions, and generalizability across age groups with a large sample size.

This study found that adding cutaneous sound data to conventional masseter EMG can improve the accuracy of detecting bruxism‐like movements. Although challenges remain in accurately identifying clenching and bracing, combining methods such as masseter muscle EMG and sound data could be valuable for measuring the mechanical load on the teeth. This highlights the negative effects of bruxism on dental health. Ultimately, this approach can lead to a better understanding of how common tooth‐damaging bruxism is, support effective management strategies, and help develop new techniques to prevent tooth loss caused by bruxism.

## 5. Conclusion

Machine‐learning‐based EMG discrimination demonstrated high accuracy in differentiating simulated bruxism from non‐BMs. Among the biological sources, the masseter muscle showed the highest sensitivity, specificity, and predictive values in single‐stream HMM, while the multi‐stream model achieved even greater accuracy than the single‐stream model. These findings indicate that machine‐learning‐based assessments can offer high discrimination accuracy even with a single EMG source, and that adding cutaneous sound data may serve as a complementary biological input to further enhance bruxism detection accuracy.

## Ethics Statement

All procedures performed in this study involving human participants were in accordance with the ethical standards of the institutional and/or national research committee and with the 1964 Helsinki declaration and its later amendments or comparable ethical standards.

## Consent

Informed consent was obtained from all the participants included in the study.

## Conflicts of Interest

The authors declare no conflicts of interest.

## Funding

This study was funded by the Ministry of Education, Science and Culture, Japan (Grant‐in‐Aid for Young Investigatory Research No. 20K23107) and Ko Omori received this research fund.

## Data Availability

The data that support the findings of this study are available from the corresponding author upon reasonable request.
